# Finerenone in heart failure and chronic kidney disease with type 2 diabetes: FINE-HEART pooled analysis of cardiovascular, kidney and mortality outcomes

**DOI:** 10.1038/s41591-024-03264-4

**Published:** 2024-09-01

**Authors:** Muthiah Vaduganathan, Gerasimos Filippatos, Brian L. Claggett, Akshay S. Desai, Pardeep S. Jhund, Alasdair Henderson, Meike Brinker, Peter Kolkhof, Patrick Schloemer, James Lay-Flurrie, Prabhakar Viswanathan, Carolyn S. P. Lam, Michele Senni, Sanjiv J. Shah, Adriaan A. Voors, Faiez Zannad, Peter Rossing, Luis M. Ruilope, Stefan D. Anker, Bertram Pitt, Rajiv Agarwal, John J. V. McMurray, Scott D. Solomon

**Affiliations:** 1 Brigham and Women’s Hospital, Harvard Medical School, Boston, MA USA; 2 National and Kapodistrian University of Athens, School of Medicine, Attikon University Hospital, Athens, Greece; 3 University of Glasgow, Glasgow, UK; 4 Bayer, Research & Development, Pharmaceuticals, Berlin, Germany; 5 National Heart Centre Singapore & Duke-National University of Singapore, Singapore, Singapore; 6 University of Milano-Bicocca, Papa Giovanni XXIII Hospital, Bergamo, Italy; 7 Northwestern University Feinberg School of Medicine, Chicago, IL USA; 8 University of Groningen, Groningen, The Netherlands; 9 University of Lorraine, Nancy, France; 10 Steno Diabetes Center Copenhagen and University of Copenhagen, Copenhagen, Denmark; 11 Hospital 12 de Octubre, Madrid, Spain; 12 Charité University, Berlin, Germany; 13 University of Michigan, Ann Arbor, MI USA; 14 Indiana University School of Medicine, Indianapolis, IN USA

**Keywords:** Heart failure, Chronic kidney disease, Diabetes

## Abstract

Cardiovascular-kidney-metabolic syndrome is an emerging entity that connects cardiovascular diseases, chronic kidney disease and diabetes. The non-steroidal mineralocorticoid receptor antagonist finerenone has been studied in three prospective randomized clinical trials of patients with cardiovascular-kidney-metabolic syndrome: FIDELIO-DKD, FIGARO-DKD and FINEARTS-HF. In light of the strong epidemiological overlap and shared mechanistic drivers of clinical outcomes across cardiovascular-kidney-metabolic syndrome, we summarize the efficacy and safety of finerenone on cardiovascular, kidney and mortality outcomes in this pre-specified participant-level pooled analysis. The three trials included 18,991 participants (mean age 67 ± 10 years; 35% women). During 2.9 years of median follow-up, the primary outcome of cardiovascular death occurred in 421 (4.4%) participants assigned to finerenone and 471 (5.0%) participants assigned to placebo (hazard ratio (HR): 0.89; 95% confidence interval (CI): 0.78–1.01; *P* = 0.076). Death from any cause occurred in 1,042 (11.0%) participants in the finerenone arm and in 1,136 (12.0%) participants in the placebo arm (HR: 0.91; 95% CI: 0.84–0.99; *P* = 0.027). Finerenone further reduced the risk of hospitalization from heart failure (HR: 0.83; 95% CI: 0.75–0.92; *P* < 0.001) and the composite kidney outcome (HR: 0.80; 95% CI: 0.72–0.90; *P* < 0.001). While in this pooled analysis the reduction in cardiovascular death was not statistically significant, finerenone reduced the risks for deaths of any cause, cardiovascular events and kidney outcomes. PROSPERO identifier: CRD42024570467.

## Abstract (web summary)

This participant-level pooled analysis of the three phase 3 trials that have tested the non-steroidal mineralocorticoid receptor antagonist finerenone in patients with heart failure and chronic kidney disease with type 2 diabetes provides an integrated view of the mortality, cardiovascular and renal effects of this treatment.

## Main

Cardiovascular diseases, chronic kidney disease (CKD) and metabolic conditions frequently occur in the same individual and may share common pathophysiological pathways of disease onset and progression^1–3^. This cardiovascular-kidney-metabolic (CKM) overlap is increasingly recognized, and there is potential for individual therapies to simultaneously improve multiple adjacent disease states^4^. Activation of the mineralocorticoid receptor is a well-recognized driver of systemic and target organ inflammation and fibrosis in these disease states^5–7^. Steroidal mineralocorticoid receptor antagonists, such as spironolactone and eplerenone, target these shared pathways, but their widespread use has been limited, especially in patients with CKM multimorbidity^8^. Gaps in implementation of steroidal mineralocorticoid receptor antagonists in heart failure (HF) are, in part, related to safety concerns due to hyperkalemia and worsening kidney function^9^.

Finerenone is a selective and potent non-steroidal mineralocorticoid receptor antagonist^5,10–13^ that may have lower risks of hyperkalemia and worsening kidney function compared to spironolactone^14^. Finerenone has been shown to reduce the risk of cardiovascular events and kidney failure in patients with CKD with type 2 diabetes (T2D)^15,16^ and has recently been shown to reduce worsening HF events in patients with HF with mildly reduced or preserved ejection fraction^17^. However, none of these trials was individually powered to evaluate treatment effects on less frequent cardiovascular-kidney outcomes, such as cardiovascular death or efficacy in key subgroups, including those with overlapping CKM conditions. A previous pooled analysis of the CKD with T2D trials showed that finerenone reduced major adverse cardiovascular events by 14% and a kidney composite outcome by 22% but remained underpowered in the evaluation of mortality outcomes^18^. Broadening this pooled population to encompass participants from the recently completed FINEARTS-HF trial will enhance precision on a range of safety and efficacy outcomes and allow evaluation of previously understudied subpopulations.

In light of the strong epidemiological overlap and shared mechanistic drivers, pooled integrated assessment of these CKM trials was pre-specified in a participant-level analysis of three phase 3 global, multicenter, double-blind, placebo-controlled randomized clinical trials of finerenone (FINE-HEART).

## Results

Overall, FINE-HEART comprised 18,991 participants from these three trials. Baseline clinical profiles and treatment patterns are summarized for the overall pooled population (Table 1) and by individual trial (Extended Data Table 4). Mean age was 67 ± 10 years; 35.1% were women; and participants were enrolled across all major geographic regions. Participants were at high risk for CKD progression (Extended Data Fig. 2) with either reduced estimated glomerular filtration rate (eGFR) (30.1% with eGFR < 45 and 26% with eGFR 45–60 ml min^−1^ 1.73 m^−^^2^) and/or albuminuria (30.8% with ‘A2’ urine albumin creatinine ratio (UACR) 30–299 mg g^−1^ and 49.2% with ‘A3’ UACR ≥ 300 mg g^−1^). In total, 2,307 (12.1%) participants had all CKM conditions (HF, CKD and diabetes) (Extended Data Fig. 3). At baseline, 1,690 (8.9%) participants were co-treated with a sodium–glucose co-transporter-2 (SGLT2) inhibitor, and 1,110 (5.8%) participants were co-treated with a glucagon-like peptide-1 receptor agonist (GLP-1RA). Baseline characteristics and concurrent medical management were well balanced between treatment arms (Table 1). Most participants were titrated to a final dose of 20 mg (4,645 (69.8%) in the placebo arm and 4,248 (63.6%) in the finerenone arm), and some (exclusively in FINEARTS-HF) were titrated to 40 mg (920 (13.8%) in the placebo arm and 832 (12.5%) in the finerenone arm).

**Table 1:** Baseline characteristics

	Finerenone	Placebo
	*n* = 9,501	*n* = 9,490
Age	67.0 ± 10.0	67.1 ± 10.2
Female	3,390 (35.7%)	3,274 (34.5%)
Race^a^		
Asian	1,910 (20.1%)	1,946 (20.5%)
Black	300 (3.2 %)	308 (3.2 %)
Other	476 (5.0 %)	447 (4.7 %)
White	6,815 (71.7%)	6,789 (71.5%)
Region		
Asia	1,808 (19.0%)	1,815 (19.1%)
Eastern Europe	3,001 (31.6%)	2,941 (31.0%)
Latin America	1,041 (11.0%)	1,034 (10.9%)
North America	1,259 (13.3%)	1,261 (13.3%)
Western Europe, Oceania and Others	2,392 (25.2%)	2,439 (25.7%)
Body mass index (kg m^-2^)	30.9 ± 6.1	30.9 ± 6.0
Systolic blood pressure (mmHg)	134.5 ± 14.9	134.4 ± 15.0
Potassium (mmol L^-1^)	4.4 ± 0.5	4.4 ± 0.5
eGFR (mL min^-1^ 1.73 m^-2^)	58.9 ± 21.0	59.1 ± 21.3
eGFR category		
<25 mL min^-1^ 1.73 m^-2^	100 (1.1%)	94 (1.0%)
25 to <45 mL min^-1^ 1.73 m^-2^	2,742 (28.9%)	2,782 (29.3%)
45 to <60 mL min^-1^ 1.73 m^-2^	2,513 (26.5%)	2,469 (26.0%)
≥60 mL min^-1^ 1.73 m^-2^	4,145 (43.6%)	4,143 (43.7%)
UACR (mg g^-1^)	283 (46–836)	293 (47–855)
Albuminuria category		
A1 (<30 mg g^-1^)	1,885 (20.1%)	1,856 (19.8%)
A2 (30 to <300 mg g^-1^)	2,910 (31.0%)	2,883 (30.7%)
A3 (≥300 mg g^-1^)	4,602 (49.0%)	4,646 (49.5%)
Hemoglobin A1c (%)	7.3 ± 1.4	7.3 ± 1.4
AF on electrocardiogram	1,449 (15.3%)	1,379 (14.5%)
History of HF^b^	3,488 (36.7%)	3,520 (37.1%)
Baseline CKD^c^	7,949 (83.7%)	7,929 (83.6%)
History of DM^d^	7,715 (81.2%)	7,714 (81.3%)
Background medication use		
Diuretics	6,291 (66.2%)	6,340 (66.8%)
ACEi/ARB/ARNI	8,866 (93.3%)	8,860 (93.4%)
Aspirin	4,145 (43.6%)	4,171 (44.0%)
Statins	6,687 (70.4%)	6,750 (71.1%)
SGLT-2 inhibitors	829 (8.7%)	861 (9.1%)
GLP-1 receptor agonists	576 (6.1%)	534 (5.6%)
Potassium-lowering therapies^e^	99 (1.0%)	96 (1.0%)

A1, A2 and A3, albuminuria categories; ACEi, angiotensin-converting enzyme inhibitors; AF, atrial fibrillation; ARB, angiotensin II receptor blocker; ARNI, angiotensin receptor neprilysin inhibitor; DM, diabetes mellitus.

^a^Represents self-reported race. Participants choosing not to disclose race or who self-identified as multiple races are included in the ‘Other’ category for descriptive purposes.

^b^HF includes all participants in FINEARTS-HF and those with investigator-reported history of HF in the primary CKD outcomes trials (FIDELIO-DKD and FIGARO-DKD).

^c^CKD includes all participants in the primary CKD outcomes trials (FIDELIO-DKD and FIGARO-DKD) and participants in FINEARTS-HF with baseline eGFR of <60 ml min^−1^ 1.73 m^−2^.

^d^Diabetes includes all participants in the primary CKD outcomes trials (FIDELIO-DKD and FIGARO-DKD) and those with a history of diabetes in FINEARTS-HF.

^e^Includes patiromer, sodium polystyrene sulfonate and calcium polystyrene sulfonate.

**Fig. 1:**
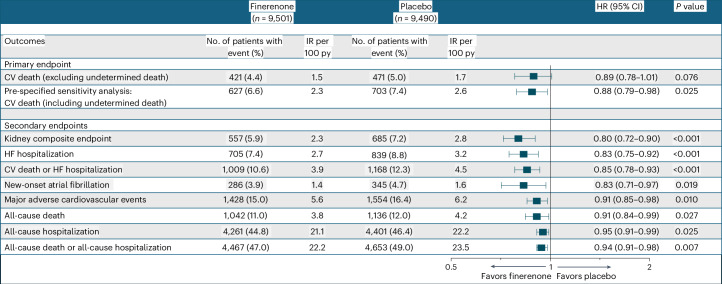
Efficacy outcomes. The primary efficacy outcome was cardiovascular death (deaths of undetermined causes were excluded). Pre-specified sensitivity analysis of the primary efficacy outcome considered deaths of undetermined causes as having a cardiovascular cause. The kidney composite outcome was defined as a sustained decrease in eGFR to ≥50% from baseline, sustained decline in eGFR to <15 ml min^−1^ 1.73 m^−^^2^, kidney failure and death due to kidney failure. Major adverse cardiovascular events included cardiovascular death or a non-fatal cardiovascular event (HF hospitalization, myocardial infarction or stroke). The composite of all-cause death or all-cause hospitalization was defined post hoc. All primary and secondary outcomes were analyzed as time to first outcomes using a stratified Cox proportional hazards model including the study intervention group as a fixed effect and stratified by geographic region and individual trial. All two-sided *P* values are reported without adjustment for multiple comparisons. The bars represent 95% CIs around each treatment effect point estimate. CV, cardiovascular; IR, incidence rate.

**Fig. 2:**
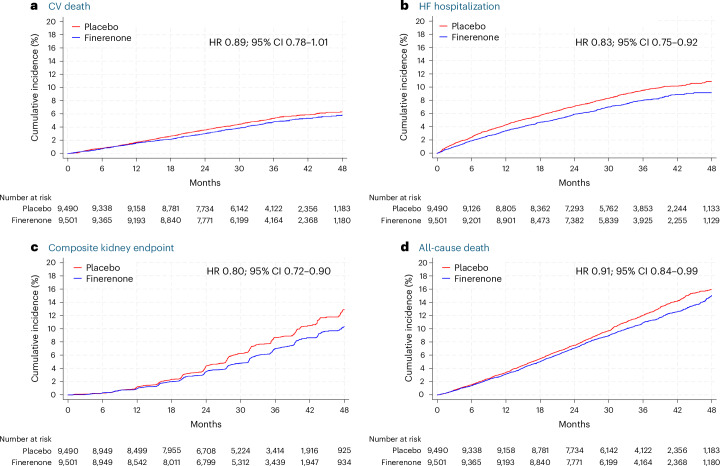
Cumulative incidence of key efficacy outcomes. Incidence of cardiovascular (CV) death (primary outcome) (**a**); HF hospitalization (**b**); kidney composite outcome (sustained decrease in eGFR to ≥50% from baseline, sustained decline in eGFR to <15 ml min^−1^ 1.73 m^−^^2^, kidney failure and death due to kidney failure) (**c**); and all-cause death (**d**).

Median duration of follow-up was 2.6 years (FIDELIO-DKD), 3.4 years (FIGARO-DKD) and 2.6 years (FINEARTS-HF). Median follow-up of the pooled patient population was 2.9 years. The primary endpoint of cardiovascular death occurred in 421 (4.4%) participants in the finerenone arm and in 471 (5.0%) participants in the placebo arm (hazard ratio (HR): 0.89; 95% confidence interval (CI): 0.78–1.01; *P* = 0.076) with consistent findings in pre-specified sensitivity analysis including both cardiovascular deaths and undetermined deaths (6.6% versus 7.4%; HR: 0.88; 95% CI: 0.79–0.98; *P* = 0.025) (Fig. 1). Effects on cardiovascular death were consistent across individual trials: FIDELIO-DKD (HR: 0.90; 95% CI: 0.65–1.23); FIGARO-DKD (HR: 0.81; 95% CI: 0.62–1.04); and FINEARTS-HF (HR: 0.93; 95% CI: 0.78–1.11); *P*_interaction_ = 0.68 (Extended Data Fig. 4). Deaths from any cause occurred in 1,042 (11.0%) participants in the finerenone arm and in 1,136 (12.0%) participants in the placebo arm (HR: 0.91; 95% CI: 0.84–0.99; *P* = 0.027) (Fig. 2). A complete accounting of cause-specific death by treatment arm is shown in Table 2.

**Table 2:** Cause-specific death

Cause of death	Finerenone		Placebo		Total	
	*n*	Rate per 100 py	*n*	Rate per 100 py	*n*	Rate per 100 py
All-cause death	1,042	3.8	1,136	4.2	2,178	4.0
Cardiovascular causes						
Sudden	188	0.7	230	0.9	418	0.8
Heart failure	92	0.3	113	0.4	205	0.4
Stroke	47	0.2	59	0.2	106	0.2
Myocardial infarction	35	0.1	37	0.1	72	0.1
Cardiovascular procedural	12	0.0	7	0.0	19	0.0
Other cardiovascular related	47	0.2	25	0.1	72	0.1
Non-CV causes						
Infection	194	0.7	188	0.7	382	0.7
Malignancy	121	0.4	149	0.6	270	0.5
Renal	4	0.0	6	0.0	10	0.0
Other non-cardiovascular related	96	0.3	90	0.3	186	0.3
Undetermined	206	0.8	232	0.9	438	0.8

py, patient years.

Finerenone reduced the risk of the composite kidney outcome whether defined inclusive of a sustained decrease in eGFR to ≥50% from baseline (HR: 0.80; 95% CI: 0.72–0.90; *P* < 0.001) or a sustained decrease in eGFR to ≥57% from baseline (HR: 0.79; 95% CI: 0.70–0.91; *P* < 0.001). These kidney effects appeared to be driven by FIDELIO-DKD and FIGARO-DKD (Extended Data Fig. 4). The incidences of the individual components of the kidney composite endpoint are displayed in Extended Data Table 5. Finerenone reduced the risk of hospitalizations due to HF alone (HR: 0.83; 95% CI: 0.75–0.92; *P* < 0.001) and the composite of cardiovascular death or HF hospitalization (HR: 0.85; 95% CI: 0.78–0.93; *P* < 0.001). Hospitalizations of any cause were also lower with finerenone compared to placebo (HR: 0.95; 95% CI: 0.91–0.99; *P* = 0.025). Finerenone further reduced the risk of the composite of all-cause death or all-cause hospitalization (HR: 0.94; 95% CI: 0.91–0.98; *P* = 0.007). Additional risk reductions were observed for the prevention of new-onset atrial fibrillation and major adverse cardiovascular events (Fig. 1).

**Fig. 3:**
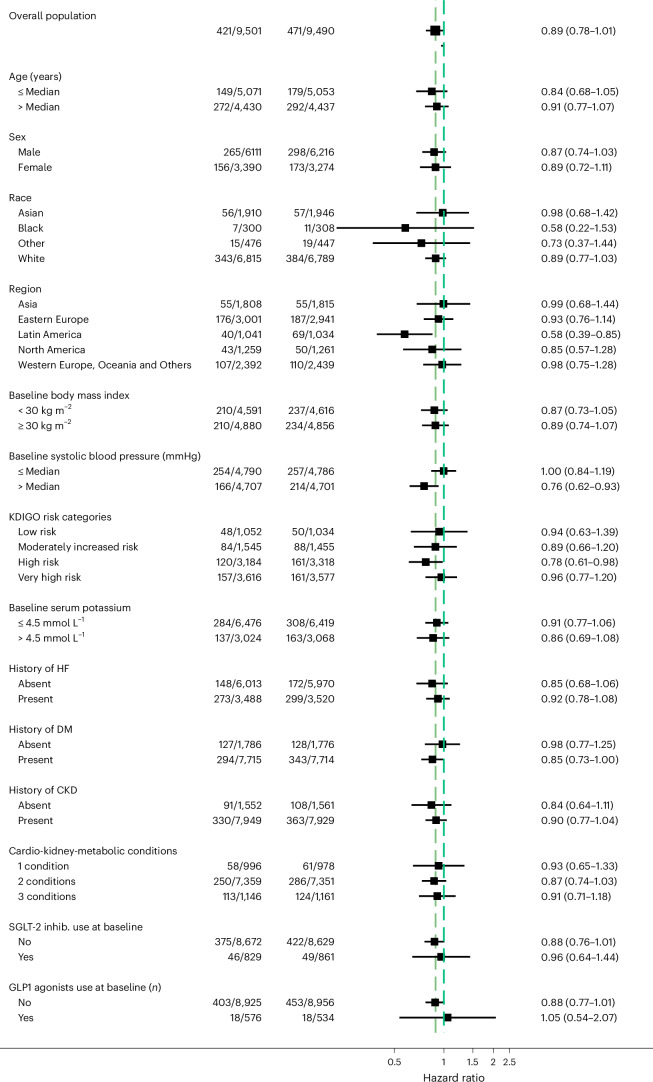
Subgroup forest plot for primary outcome (CV death). The median age was 68 years, and the median systolic blood pressure was 135 mmHg. For each patient, the presence of CKM conditions was summed for the following comorbidities: CKD, HF and/or DM. The bars represent 95% CIs around each treatment effect point estimate. CV, cardiovascular; DM, diabetes mellitus.

Treatment effects on cardiovascular death were generally consistent across the 16 subgroups examined (Fig. 3). The efficacy of finerenone on cardiovascular death was consistent across the range of eGFR (*P*_interaction_ = 0.32) and UACR (*P*_interaction_ = 0.55) (Extended Data Fig. 5). Treatment effects on cardiovascular death were also consistent across a range of CKM disease burden: one condition (HR: 0.93; 95% CI: 0.65–0.1.33); two conditions (HR: 0.87; 95% CI: 0.74–1.03); and three conditions (HR: 0.91; 95% CI: 0.71–1.18); *P*_interaction_ = 0.94.

Incidences of any serious adverse event were lower with finerenone than placebo (34.6% versus 36.6%), although incidences of serious adverse events leading to drug discontinuation were slightly higher with finerenone (5.4% versus 4.6%). Laboratory-defined hyperkalemia was increased, whereas laboratory-defined hypokalemia was decreased, with finerenone. Incidences of investigator-reported hyperkalemia leading to permanent treatment discontinuation (1.3% versus 0.5%) and hyperkalemia-related hospitalization (0.8% versus 0.2%) were higher with finerenone. There were no deaths related to hyperkalemia and no between-group differences in incidences of acute kidney injury (Table 3).

**Table 3:** Safety outcomes

	Finerenone	Placebo
	*n* = 9,482	*n* = 9,467
Any serious adverse event	3,283 (34.6%)	3,463 (36.6%)
Any adverse event leading to treatment discontinuation	515 (5.4 %)	434 (4.6 %)
Any potassium >5.5 mmol L^−1 (^^a)^	1,535 (16.5%)	714 (7.7 %)
Any potassium >6.0 mmol L^−1 (^^a)^	311 (3.3 %)	133 (1.4 %)
Any potassium <3.5 mmol L^−1 (^^a)^	448 (4.8 %)	938 (10.1%)
Hyperkalemia^b^	1,216 (12.8%)	586 (6.2 %)
Hyperkalemia leading to treatment discontinuation^b^	123 (1.3 %)	43 (0.5 %)
Hyperkalemia leading to hospitalization^b^	80 (0.8 %)	17 (0.2 %)
Hyperkalemia leading to death^b^	0 (0.0 %)	0 (0.0 %)
Acute kidney injury	345 (3.6 %)	316 (3.3 %)
Acute kidney injury leading to treatment discontinuation	15 (0.2 %)	12 (0.1 %)
Acute kidney injury leading to hospitalization	143 (1.5 %)	116 (1.2 %)
Systolic blood pressure < 100 mmHg	1,040 (11.1%)	651 (7.0%)
Gynecomastia or breast hyperplasia	16 (0.2%)	19 (0.2%)

Treatment-emergent adverse events are defined as any adverse event occurring in any patient who has received at least one dose of study drug and within 3 d of permanent discontinuation. This safety table includes one patient who was randomized to placebo but who actually received finerenone.

^a^Based on central laboratory measurements of potassium levels.

^b^Based on investigator-reported adverse events.

## Discussion

This participant-level pooled analysis, FINE-HEART, represents, to our knowledge, the largest analysis of the efficacy and safety of the non-steroidal mineralocorticoid receptor antagonist finerenone across the CKM spectrum. Although this pooled analysis did not demonstrate a significant reduction in the primary outcome of cardiovascular death, this result was sensitive to the definition of cardiovascular death based on the classification of deaths of undetermined causes. As such, we placed greater confidence in the outcome of all-cause death, which was reduced with finerenone with nominal significance. This mortality signal with finerenone was further substantiated by clinically relevant benefits observed across a broad range of other cardiovascular-kidney outcomes, including kidney disease progression, HF hospitalizations, all-cause hospitalizations, new-onset atrial fibrillation and major adverse cardiovascular events. Treatment effects were consistent across all tested clinical subgroups, including those with multiple, intersecting CKM conditions. No new or unexpected safety signals were uncovered in this pooled analysis with a well-characterized modestly higher risk of hyperkalemia but overall lower incidences of serious adverse events and no excess risk of acute kidney injury with finerenone. Taken together, these data suggest the potential of finerenone to prevent or delay morbidity and mortality across a wide range of CKM conditions while being safe and well tolerated.

Finerenone is approved for use in patients with CKD and T2D. Although several major multi-specialty guidelines strongly recommend finerenone to delay CKD progression and prevent HF events in people with CKD with T2D^19–21^, the latest Kidney Disease: Improving Global Outcomes (KDIGO) guideline^22^ has offered a class 2A recommendation potentially related to residual uncertainties regarding the mortality effects of finerenone in the FIDELIO-DKD and FIGARO-DKD trials. Furthermore, according to the guideline, its use is to be considered in patients already on standard-of-care therapies, such as maximally tolerated renin–angiotensin system inhibitors and/or SGLT2 inhibitors. These pooled data did not identify heterogeneity with finerenone’s effects on cardiovascular death by background use of SGLT2 inhibitors or GLP-1RA, although the statistical power of these subgroup findings was limited. This pooled analysis bolsters recent calls for the combination use of finerenone alongside these therapies as foundational ‘pillars’ of care to maximize improvements in cardiovascular-kidney outcomes^23,24^; additional evidence will be needed to inform the expected efficacy and safety of various combinations of risk-lowering therapies.

Finerenone was shown to robustly reduce the kidney composite outcome by 20% in this pooled analysis, driven by benefits observed in FIDELIO-DKD and FIGARO-DKD. FINEARTS-HF enrolled a primary HF population with some co-existing kidney disease but with relatively low levels of albuminuria; therefore, CKD progression over a relatively short period of follow-up was difficult to evaluate. It is reassuring that finerenone did not show an increase in reports of acute kidney injury, despite the high baseline kidney risk profile of the patient population and the varied clinical care settings of therapeutic initiation.

Although each trial had broad eligibility criteria, some groups were understudied in individual trials. For instance, both FIGARO-DKD and FIDELIO-DKD exclusively enrolled participants with CKD and T2D with albuminuria. FINEARTS-HF provides complementary evidence related to finerenone’s therapeutic effects in previously understudied populations, including those without diabetes (~60% of trial enrollment), those without CKD and those without significant urinary albumin excretion (>60% of the trial with UACR <30 mg g^−1^). FINEARTS-HF exclusively enrolled patients with symptomatic HF across clinical care settings, whereas FIDELIO-DKD and FIGARO-DKD specifically excluded patients with symptomatic HF with reduced ejection fraction. It is noteworthy that FIDELIO-DKD and FIGARO-DKD did enroll 5–10% of patients with HF with mildly reduced or preserved ejection fraction, emphasizing the overlap across these trials. These pooled data demonstrate that finerenone’s benefits extend to patients with different degrees of CKM multimorbidity and across broad patient profiles and enhance precision of estimates of risk reductions beyond previous analyses^18^. The diverse spectrum of benefits on HF, arrhythmia, atherosclerotic risk and kidney disease progression also underscores the systemic actions of finerenone in attenuating the adverse multi-organ effects of mineralocorticoid receptor overactivation^5^.

A key strength of this pooled analysis was access to individual participant-level data from all phase 3 trials conducted to date with finerenone, which allowed harmonization of data elements related to baseline clinical characteristics, outcomes and subgroups. Major efficacy outcomes were independently adjudicated, and safety assessments were conducted with aligned definitions in a standardized fashion. As undetermined deaths are variably handled across trials of CKM conditions, including in the three trials examined in this pooled study, separate analyses were carried out for cardiovascular death excluding and including these deaths.

This pooled analysis is subject to several limitations. The findings were derived from randomized clinical trials with specific inclusion and exclusion criteria and, thus, may not be generalizable to all populations treated in clinical practice. Despite the large global population studied, enrollment of select groups, such as Black patients, remained limited. Certain data elements were not consistently available across trials to allow for pooling. For instance, urgent HF visits, which were included as a part of the FINEARTS-HF primary outcome, were not collected in FIDELIO-DKD and FIGARO-DKD. Background use of SGLT2 inhibitors and GLP-1RA was only modest, limiting firm conclusions of the additive effects of finerenone. No adjustment was made for testing of multiple comparisons. Finally, not all trials contributed equally to each of the subgroups examined; for instance, only FINEARTS-HF included patients who did not have diabetes.

While in this pooled analysis the reduction in cardiovascular death was not statistically significant, finerenone reduced the risks for deaths of any cause, cardiovascular events and kidney outcomes while being safe and well tolerated. The totality of the evidence thus supports the disease-modifying potential of finerenone in broad, high-risk patient populations encompassing cardiovascular, kidney and metabolic diseases.

## Methods

### Search strategy and trial selection

**Extended Data Fig. 1:**
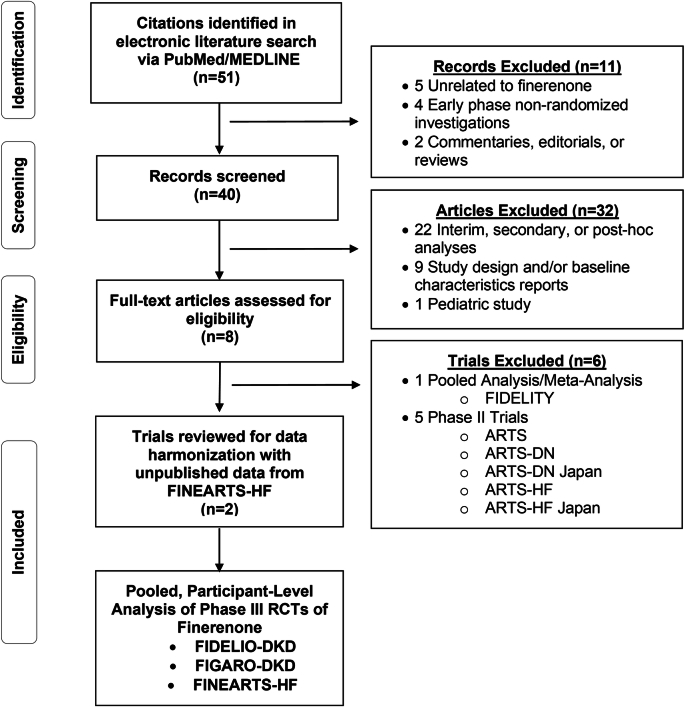
Systematic Search of the Literature to Identify Eligible Trials for Inclusion in FINE-HEART. We conducted a systematic review of the literature using PubMed and MEDLINE to ensure that relevant trials were not missed to be included in this pooled analysis.

**Extended Data Fig. 2:**
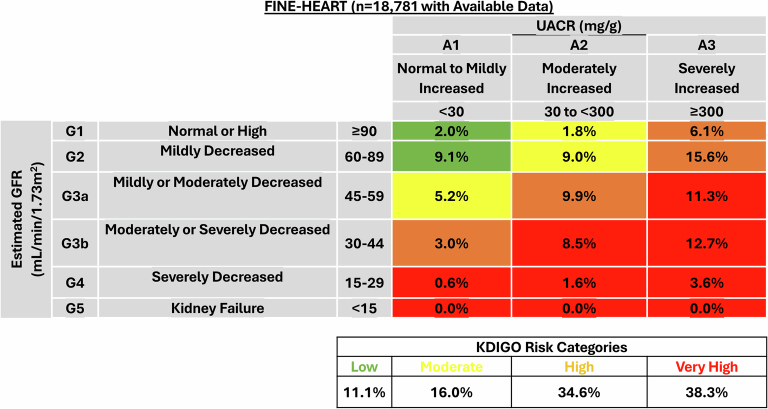
KDIGO Risk Distribution at Baseline in FINE-HEART. Participants were at high risk for chronic kidney disease progression with either reduced estimated glomerular filtration rate or elevated levels of urine albumin creatinine ratio.

**Extended Data Fig. 3:**
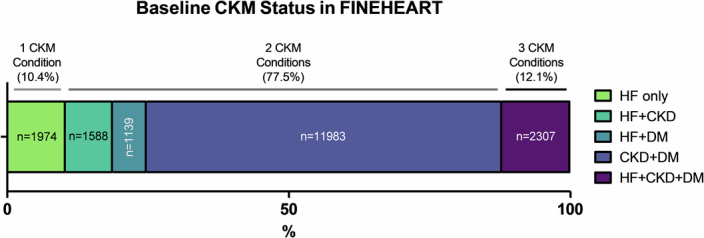
Baseline Cardio-Kidney-Metabolic Overlap in FINE-HEART. Participants included in this pooled analysis had a high degree of cardio-kidney-metabolic overlap with 90% having at least 2 conditions (heart failure, chronic kidney disease, or diabetes).

**Extended Data Fig. 4:**
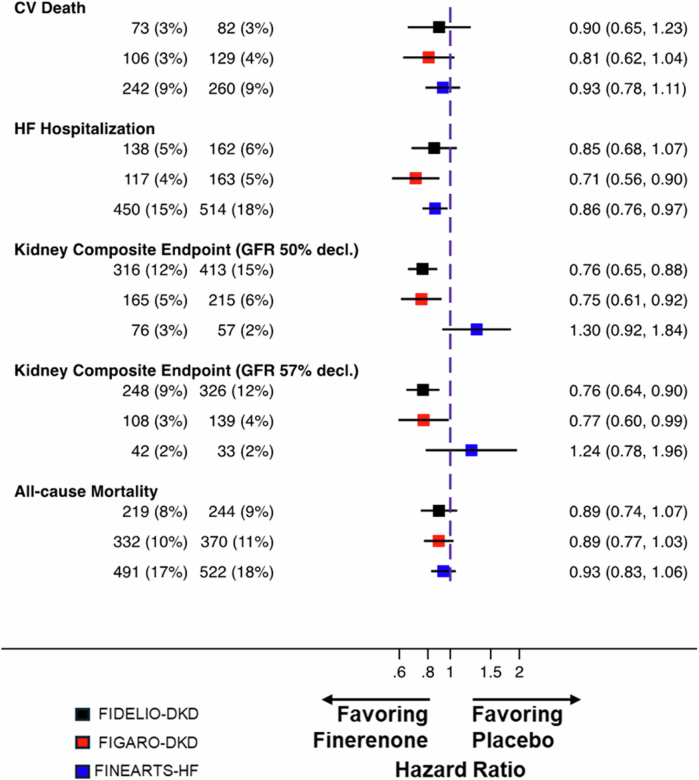
Key Efficacy Outcomes in Each Individual Trial. The bars represent 95% confidence intervals around each treatment effect point estimate.

**Extended Data Fig. 5:**
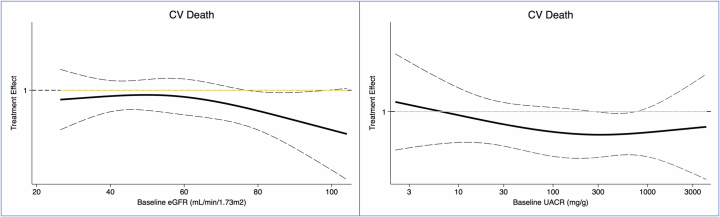
Treatment Effect on Primary Outcome of CV Death Across Baseline eGFR (Left Panel) and UACR (Right Panel). The solid line represents the treatment effect estimate and the dashed lines represent 95% confidence intervals. Abbreviations: CV = cardiovascular; eGFR = estimated glomerular filtration rate; UACR = urine albumin creatinine ratio.

We conducted a participant-level pooled analysis of two trials of CKD and T2D (FIDELIO-DKD (Finerenone in Reducing Kidney Failure and Disease Progression in Diabetic Kidney Disease; NCT02540993) and FIGARO-DKD (Finerenone in Reducing Cardiovascular Mortality and Morbidity in Diabetic Kidney Disease; NCT02545049)) and a trial of patients with HF (FINEARTS-HF (FINerenone trial to investigate Efficacy and sAfety superioR to placebo in paTientS with Heart Failure; NCT04435626)) that included patients with and without diabetes. The designs^25–27^ and primary results^15–17^ of each of the three trials have been published. Key design elements of each of the trials are summarized in Extended Data Table 1. We further conducted a systematic review of the literature using PubMed and MEDLINE to ensure that other relevant trials were not missed. We searched for randomized clinical trials of finerenone published from database inception to 24 July 2024. The following string was used in the PubMed/MEDLINE pre-specified search query to identify potential studies to be included in the meta-analysis:

(‘finerenone’[Extended Data Concept] OR ‘finerenone’[All Fields]) AND (randomizedcontrolledtrial[Filter]). The pre-specified search query, which was run on 24 July 2024, did not identify any additional phase 3 trials that met criteria for inclusion (Extended Data Fig. 1). Data from the FINEARTS-HF trial were unpublished at the time of analysis and were included with permission from the steering committee and trial sponsor.

### Design of FIDELIO-DKD and FIGARO-DKD

In brief, both FIDELIO-DKD and FIGARO-DKD trials enrolled adults (≥18 years) with T2D and CKD across 48 countries. FIDELIO-DKD required a UACR of 30 to <300 mg g^−1^, an eGFR of 25 to <60 ml min^−1^ 1.73 m^−2^ and a history of diabetic retinopathy or a UACR of 300–5,000 mg g^−1^ and an eGFR of 25 to <75 ml min^−1^ 1.73 m^−^^2^. FIGARO-DKD required a UACR of 30 to <300 mg g^−1^ and an eGFR of 25–90 ml min^−1^ 1.73 m^−^^2^ or a UACR of 300–5,000 mg g^−1^ and an eGFR of ≥60 ml min^−1^ 1.73 m^−^^2^. Both trials required a serum potassium level of ≤4.8 mmol L^−1^ for enrollment. Renin–angiotensin system inhibitor use and dosing were optimized before screening during run-in phases (lasting 4–16 weeks) in both trials. Patients with symptomatic HF with reduced ejection fraction were excluded, but those with HF and higher ejection fraction were eligible.

### Design of FINEARTS-HF

FINEARTS-HF enrolled adults (≥40 years) with symptomatic HF with mildly reduced or preserved ejection fraction across 37 countries. Key inclusion criteria included left ventricular ejection fraction ≥40%, elevated natriuretic peptides (adjusted based on atrial fibrillation status and clinical setting of screening), evidence of structural heart disease and recent diuretic use for at least 30 d. Patients were required to have an eGFR of ≥25 ml min^−1^ 1.73 m^−^^2^ and a serum potassium level of ≤5.0 mmol L^−1^ for enrollment. Participants could be enrolled regardless of clinical care setting (whether hospitalized, recently hospitalized or ambulatory).

All participants were randomly allocated to finerenone or placebo with initial dosing determined based on kidney function. The initial dose was 10 mg for patients with an eGFR of <60 ml min^−1^ 1.73 m^−^^2^, titrated up to a target dose of 20 mg once daily as tolerated. Participants with an eGFR of ≥60 ml min^−1^ 1.73 m^−^^2^ were started at the target dose of 20 mg once daily. In FINEARTS-HF, participants with an eGFR of >60 ml min^−1^ 1.73 m^−^^2^ were started on 20 mg and could be titrated up to 40 mg once daily as tolerated, whereas 20 mg was the target dose for patients with an eGFR of ≤60 ml min^−1^ 1.73 m^−^^2^. As dose-dependent effects of finerenone have been observed on natriuretic peptide levels in the preceding phase 2 program of patients with worsening HF^28^, FINEARTS-HF examined a higher target maintenance dose of 40 mg (in those with an eGFR of >60 ml min^−1^ 1.73 m^−2^) than in FIDELIO-DKD and FIGARO-DKD. The trial protocols were approved by ethics committees or institutional review boards at all participating sites, and all patients provided explicit written informed consent.

### FINE-HEART pooling strategy

Individual participant-level data were accessed and pooled with harmonized data elements for baseline characteristics and clinical outcomes (Extended Data Table 2). All participants randomized in each of the three trials were considered for this pooled analysis with only patients with critical Good Clinical Practice violations excluded. A total of 160 randomized patients (60 patients in FIDELIO-DKD, 85 patients in FIGARO-DKD and 15 patients in FINEARTS-HF) were prospectively excluded before database lock from all analyses because of critical Good Clinical Practice violations or due to re-randomization of the same subject. In addition, 36 participants in FIDELIO-DKD and FIGARO-DKD were confirmed to have critical Good Clinical Practice violations after database lock. These 196 participants were excluded from all efficacy and safety analysis in FINE-HEART. The final sample size was 18,991 participants.

### FINE-HEART pooled analysis outcomes

All efficacy outcomes were analyzed in randomized patients under intention-to-treat principles, and all safety outcomes were analyzed in randomized patients who had taken at least one dose of the study drug. All deaths were adjudicated by independent clinical endpoint committees in each of the respective trials included in this pooled analysis (specific adjudication criteria included in the Supplementary Methods).

The pre-specified primary outcome for FINE-HEART was time to cardiovascular death. The definition of cardiovascular death differed slightly among the three trials and was harmonized for FINE-HEART as time to cardiovascular death (excluding undetermined deaths) (Extended Data Table 2). Other pre-specified outcomes included a kidney composite outcome (defined as a sustained decrease in eGFR to ≥50% from baseline, sustained decline in eGFR to <15 ml min^−1^ 1.73 m^−^^2^, kidney failure and death due to kidney failure), HF hospitalization, composite of cardiovascular death or HF hospitalization, new-onset atrial fibrillation, major adverse cardiovascular events (a composite of non-fatal myocardial infarction, non-fatal stroke, HF hospitalization or cardiovascular death), all-cause death and hospitalization for any reason. The kidney composite endpoint inclusive of a sustained decrease in eGFR to ≥57% from baseline (corresponding to a doubling of serum creatinine) was additionally reported. The composite of all-cause death or all-cause hospitalization was defined post hoc to describe the total burden or morbidity and mortality. Select treatment-emergent adverse events related to hyperkalemia, acute kidney injury, hypotension and gynecomastia were additionally reported. The primary outcome (cardiovascular death) was assessed across key subgroups, including age, sex, race, region, baseline body mass index, baseline systolic blood pressure, KDIGO risk, baseline serum potassium levels, baseline eGFR, baseline UACR, history of HF, history of diabetes, presence of CKD, number of CKM conditions (CKD, HF and/or diabetes) and baseline use of SGLT2 inhibitors or GLP-1RA.

### Statistical analysis

All primary and secondary outcomes were analyzed as time to first outcomes using a stratified Cox proportional hazards model including the study intervention group as a fixed effect and stratified by geographic region and individual trial (Extended Data Table 2). All treatment effect estimates are presented as HRs with associated 95% CIs. Select primary and secondary outcomes were additionally graphically displayed using Kaplan–Meier methods. There was no multiple testing strategy; namely, testing for secondary outcomes continued even if results for the primary outcome were neutral or negative. A pre-specified sensitivity analysis was conducted for the primary outcome that considered deaths of undetermined causes to be cardiovascular deaths in all three trials. Treatment effects on cardiovascular death were assessed across all pre-specified subgroups. Incidence rates of cardiovascular death as a function of baseline eGFR and log-transformed UACR were estimated separately for each treatment arm using Poisson regression models, allowing for potentially nonlinear relationships using restricted cubic splines with three knots. The treatment effect of finerenone was then estimated as the ratio of these two group-specific estimates.

The statistical analysis plan for this pooled analysis was pre-specified, and the protocol was prospectively registered in the International Prospective Register of Systematic Reviews (PROSPERO CRD42024570467) before unblinding the FINEARTS-HF trial. All three trials were assessed as high quality with a low risk of bias before pooling (Extended Data Table 3). The trials included in this pooled analysis were funded by Bayer AG. Trial steering committees designed and oversaw their conduct in collaboration with the sponsor. However, the primary analyses, interpretation of the data and initial manuscript drafting were conducted independently by the academic teams. Statistical analyses were conducted using STATA version 18 software.

### Reporting summary

Further information on research design is available in the Nature Portfolio Reporting Summary linked to this article.

## Online content

Any methods, additional references, Nature Portfolio reporting summaries, source data, extended data, supplementary information, acknowledgements, peer review information; details of author contributions and competing interests; and statements of data and code availability are available at 10.1038/s41591-024-03264-4.

## Supplementary information

Supplemental Methods

Reporting Summary

## Figures and Tables

**Extended Data Table 1:**
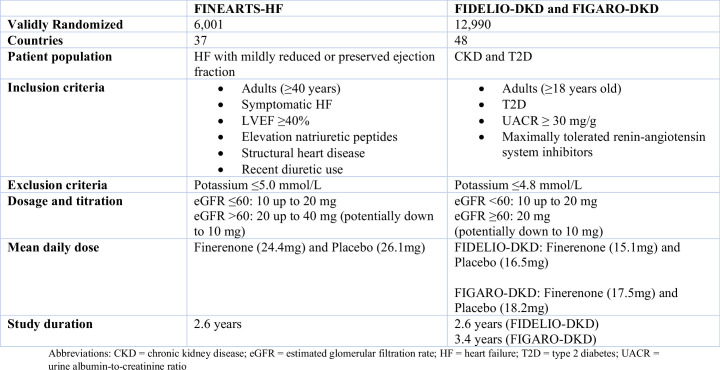
Key study design features

**Extended Data Table 2:**
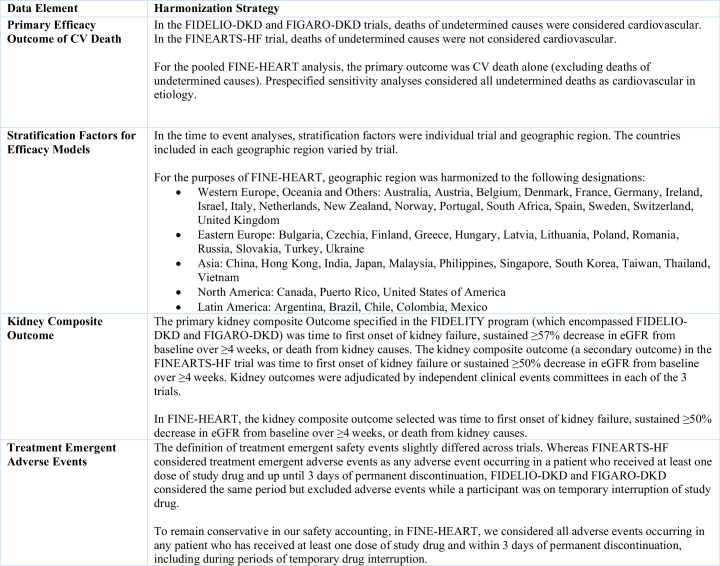
Harmonization of key outcome measures and their assessment across trials

**Extended Data Table 3:**
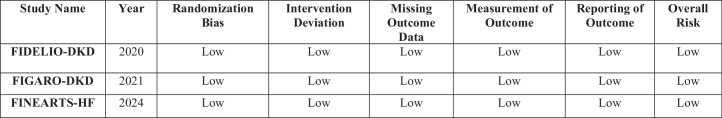
Risk of bias assessment using the revised tool to assess risk of bias in randomized trials (RoB 2.0)

**Extended Data Table 4:**
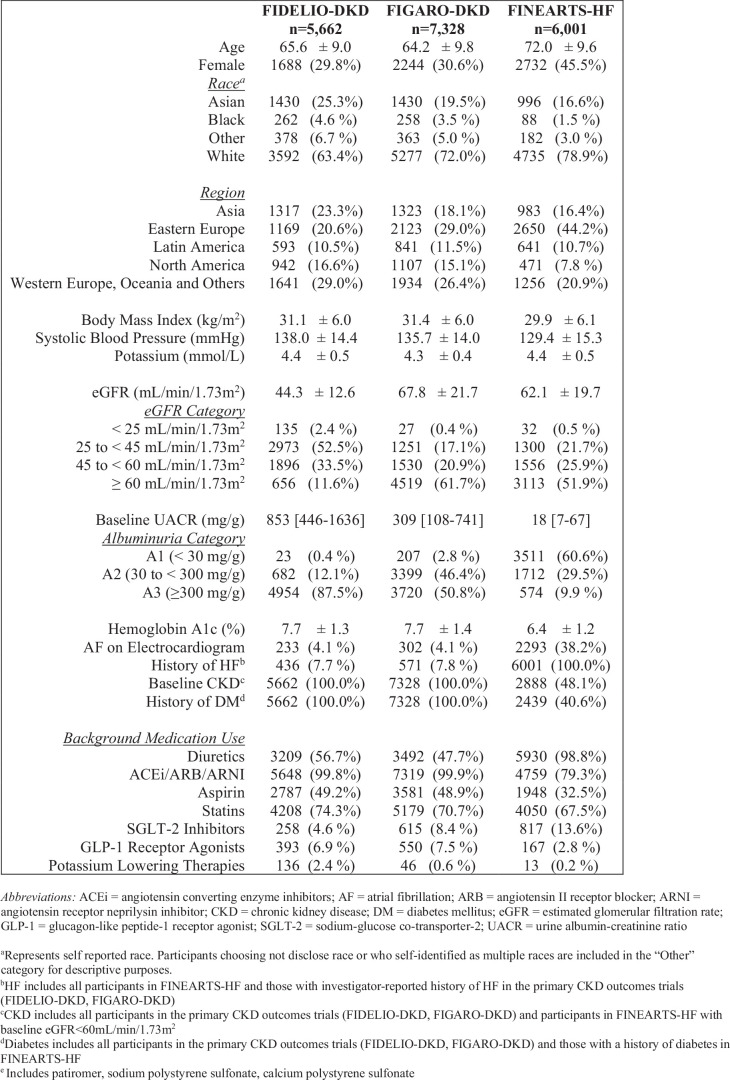
Baseline characteristics by trial

**Extended Data Table 5:**
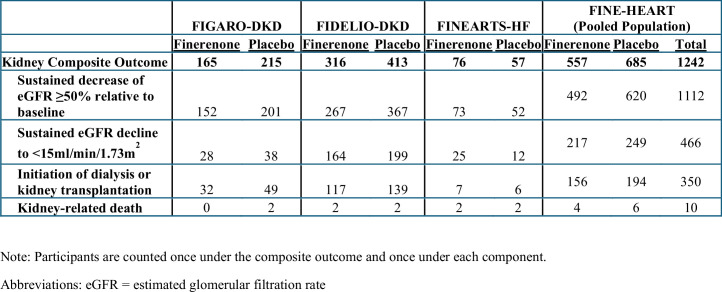
Components of the kidney composite endpoint

## Data Availability

For each of the three clinical trials (FIDELIO-DKD, FIGARO-DKD and FINEARTS-HF), Bayer (the sponsor) commits to sharing, upon reasonable request from qualified scientific and medical researchers, patient-level clinical trial data, study-level clinical trial data and protocols. Interested researchers can use https://vivli.org/ to request access to anonymized patient-level data and supporting documents from clinical studies. Data access will be granted to anonymized patient-level data, protocols and clinical study reports after approval by an independent scientific review panel, with scope and conditions laid out as on https://vivli.org/ourmember/bayer/.
