# Author Correction: Finerenone in heart failure and chronic kidney disease with type 2 diabetes: FINE-HEART pooled analysis of cardiovascular, kidney and mortality outcomes

**DOI:** 10.1038/s41591-024-03372-1

**Published:** 2024-10-28

**Authors:** Muthiah Vaduganathan, Gerasimos Filippatos, Brian L. Claggett, Akshay S. Desai, Pardeep S. Jhund, Alasdair Henderson, Meike Brinker, Peter Kolkhof, Patrick Schloemer, James Lay-Flurrie, Prabhakar Viswanathan, Carolyn S. P. Lam, Michele Senni, Sanjiv J. Shah, Adriaan A. Voors, Faiez Zannad, Peter Rossing, Luis M. Ruilope, Stefan D. Anker, Bertram Pitt, Rajiv Agarwal, John J. V. McMurray, Scott D. Solomon

**Affiliations:** 1 Brigham and Women’s Hospital, Harvard Medical School, Boston, MA USA; 2 National and Kapodistrian University of Athens, School of Medicine, Attikon University Hospital, Athens, Greece; 3 University of Glasgow, Glasgow, UK; 4 Bayer, Research & Development, Pharmaceuticals, Berlin, Germany; 5 National Heart Centre Singapore & Duke-National University of Singapore, Singapore, Singapore; 6 University of Milano-Bicocca, Papa Giovanni XXIII Hospital, Bergamo, Italy; 7 Northwestern University Feinberg School of Medicine, Chicago, IL USA; 8 University of Groningen, Groningen, The Netherlands; 9 University of Lorraine, Nancy, France; 10 Steno Diabetes Center Copenhagen and University of Copenhagen, Copenhagen, Denmark; 11 Hospital 12 de Octubre, Madrid, Spain; 12 Charité University, Berlin, Germany; 13 University of Michigan, Ann Arbor, MI USA; 14 Indiana University School of Medicine, Indianapolis, IN USA

**Keywords:** Heart failure, Chronic kidney disease, Diabetes

Correction to: *Nature Medicine* 10.1038/s41591-024-03264-4, published online 1 September 2024.

In the version of the article initially published, the first three columns of the “New-onset atrial fibrillation” row of Fig. 1 originally read “286 (3.0)”, “1.3”, “345 (3.6)” and have now been amended to “286 (3.9)”, “1.4”, “345 (4.7)” in the HTML and PDF versions of the article.

