# Supplementary material for: Finerenone in heart failure and chronic kidney disease with type 2 diabetes: FINE-HEART pooled analysis of cardiovascular, kidney and mortality outcomes
Source: Nat Med. 2024 Sep 1;30(12):3758–64. doi: 10.1038/s41591-024-03264-4 (PMC11645272; doi:10.1038/s41591-024-03264-4)
Supplement: Supplementary file 1 — Supplemental Methods [file 41591_2024_3264_MOESM1_ESM.pdf]

nature medicine

Supplementary information https://doi.org/10.1038/s41591-024-03264-4

Finerenone in heart failure and chronic kidney disease with type 2 diabetes: FINE-HEART pooled analysis of cardiovascular, kidney and mortality outcomes

In the format provided by the authors and unedited

Supplementary Methods

Cause-Specific Death Definitions Used by Adjudication Committees

FIDELIO-DKD and FIGARO-DKD

Causes of death were classified into three categories – cardiovascular death, renal death, or non-cardiovascular, and non-renal death.

Cardiovascular death

Events that were classified as cardiovascular death included the following: (1) death due to acute MI; (2) sudden cardiac death; (3) undetermined death; (4) death due to heart failure; (5) death due to stroke; (6) death due to cardiovascular procedures; or (7) death due to other cardiovascular causes.

(1) Death due to acute myocardial infarction

Death due to acute MI referred to a death by any cardiovascular mechanism (eg, arrhythmia, sudden death, heart failure, stroke, pulmonary embolus, peripheral artery disease) within 30 days after an MI and related to the immediate consequences of the MI, such as progressive heart failure or recalcitrant arrhythmia. Death that resulted from a procedure to treat a MI (including PCI and coronary artery bypass grafting), or to treat a complication resulting from MI was also considered death due to acute MI. Any death that resulted from an elective coronary procedure to treat myocardial ischaemia (ie, chronic stable angina) or any death due to an MI that occurred as a direct consequence of a cardiovascular investigation/procedure/operation was considered as a death due to a cardiovascular procedure.

(2) Sudden cardiac death

Sudden cardiac death referred to any death that occurred unexpectedly, not following an acute MI, (i.e., not within 30 days of an acute MI) and included the following deaths:

- Death witnessed and instantaneous without new or worsening symptoms
- Death witnessed within 1 hour of the onset of new or worsening cardiac symptoms, unless the symptoms suggest acute MI
- Death witnessed and attributed to an identified arrhythmia (eg, captured on an ECG recording, witnessed on a monitor, or unwitnessed but found on implantable cardioverter-defibrillator review)
- Death after unsuccessful resuscitation from cardiac arrest
- Death after successful resuscitation from cardiac arrest and without identification of a specific aetiology
- Unwitnessed death in a patient seen alive and clinically stable ≤24 hours prior to being found dead without any evidence supporting a specific non-cardiovascular cause of death

(3) Undetermined death

Any death occurring in a patient NOT observed alive within 24 hours of death and without any other likely cause of death, was recorded as undetermined. All patients who died of undetermined causes were considered by default as cardiovascular death because of the patient population and competing causes of death.

(4) Death due to heart failure

Death due to heart failure or cardiogenic shock referred to a death associated with clinically worsening symptoms and/or signs of heart failure without evidence of another cause that did NOT occur following an acute MI. Deaths due to heart failure could have various aetiologies, including single or recurrent MIs (late effect, ie, >30 days), ischaemic or non-ischaemic cardiomyopathy, hypertension, or valvular disease. Sudden deaths occurring during an admission for worsening heart failure, as well as death from progressive heart failure or cardiogenic shock, following implantation of a mechanical assist device were also classified as death due to heart failure if there was no evidence of acute MI or stroke in the previous 30 days.

(5) Death due to stroke

Any death that occurred within 30 days after a stroke was classified as death due to stroke, whether a direct consequence of the stroke or a complication of the stroke.

(6) Death due to cardiovascular procedures
Any death occurring within 30 days of a cardiovascular procedure (as a result of complications of the cardiovascular procedure) was classified as death due to cardiovascular procedures.

(7) Death due to other cardiovascular causes
Any cardiovascular death that was not included in the above categories was classified as cardiovascular death due to other causes (eg. cardiovascular haemorrhage, pulmonary embolism, or peripheral arterial disease). Non-stroke intracranial haemorrhage, non-procedural or non-traumatic vascular rupture (eg, aortic rupture) or haemorrhage causing cardiac tamponade were considered cardiovascular haemorrhages. Any other bleeding was considered as non-cardiovascular.

Renal death
Events were classified as renal death if: (1) the patient died; (2) RRT had not been initiated despite being clinically indicated; and (3) there was no other likely cause of death. If a patient was initially denied RRT for a specific reason (eg, metastatic cancer, shock, or sepsis) then another more proximal cause of death was identified.

Non-cardiovascular and non-renal death
Events were classified as non-cardiovascular and non-renal deaths if they were not thought to be due to a cardiovascular or renal cause. Non-cardiovascular and non-renal deaths were categorized as infection, malignancy, or specific other causes

The CEC will attribute cause of death according to the responsible underlying disease process rather than the immediate mechanism.

Deaths will be classified as cardiovascular, non-cardiovascular, or undetermined, and, where possible, further sub-classified as outlined below.

In light of the substantial burden of cardiorespiratory illness contributed by the COVID-19 pandemic and the potential for COVID-19 infection status to influence the adjudication of study endpoints, the CEC will in all cases report whether the death was thought to be related to COVID-19 (positive testing, typical clinical trajectory), possibly related to COVID-19 (inconclusive or absent testing, typical clinical trajectory), unrelated to COVID-19 (testing negative or not done, not suspected).

Cause-Specific Death Definitions Used by Adjudication Committees

<u>FINEARTS-HF</u>

A. Cardiovascular Death

Cardiovascular death includes death classified in any of the following categories:

1. Fatal Myocardial Infarction (MI):

Fatal MI may be adjudicated in any of the following three scenarios:

a. Death occurring within 14 days after a documented MI, in which there is no conclusive evidence of any other cause of death. Subjects who are being treated for a MI and who die as a result of complications of the MI (eg, sudden death, pump failure, or cardiogenic shock) will be classified as having had a MI-related death.

b. Autopsy evidence of a recent infarct with no conclusive evidence of any other cause of death.

c. An abrupt death that has characteristics suggestive of an acute infarct but does not meet the definition of a MI. Suggestive characteristics are:

-presentation with acute ischemic symptoms

AND one of the following:

- ECG changes indicative of an acute injury
- abnormal cardiac biomarkers

-other evidence (eg, echocardiography, ventriculography, or scintigraphy) of new ventricular wall motion abnormality

2. Heart Failure Death:

Death occurring in the context of clinically worsening symptoms and/or signs of heart failure (HF) without evidence of another cause of death.

Death occurring as a complication of the implantation of a ventricular assist device, cardiac transplant, or other surgery primarily for refractory HF.

Death occurring after referral to hospice specifically for progressive HF.

Note: If worsening HF is secondary to MI, then MI should be listed as the primary cause of death if the subject suffered an MI within 14 days of death (as above).

3. Sudden Death:

Death occurring unexpectedly in an otherwise stable subject. Further classification of sudden death will be as follows:

a. death witnessed or subject last seen alive <1 hour previously or

b. subject last seen alive ≥ 1 hr and < 24 hrs previously

4. Presumed Sudden Death

Death occurring unexpectedly in an otherwise stable subject last seen alive ≥ 24 hours previously, with circumstances suggestive of sudden death.

5. Presumed Cardiovascular Death:

Death likely due to a cardiovascular cause in which the available clinical data is insufficient to support a more specific cause of death.

6. Fatal Stroke:

Death occurring as a result of a documented stroke. Where possible, the stroke will be further classified as ischemic (non-hemorrhagic), ischemic (non-hemorrhagic) with hemorrhagic conversion, hemorrhagic, or unknown.

7. Fatal Pulmonary Embolism:

Death occurring as a direct result of a documented pulmonary embolism.

8. Cardiovascular Procedure-Related Death:

Death occurring during a cardiovascular procedure or as a result of complications related to a cardiovascular procedure (e.g. percutaneous coronary intervention), usually within 14 days. The CEC will categorize these deaths as related to percutaneous coronary intervention (PCI-related), coronary artery bypass-grafting (CABG-related), valvular procedures (valvular), or other cardiovascular procedures (other).

9. Other Cardiovascular Death:
Death resulting from a specifically documented cardiovascular cause other than those listed above.

B. Non-Cardiovascular Death
If an unequivocal and documented non-cardiovascular cause can be established as the primary cause of death, the event will be classified as non-cardiovascular. Non-cardiovascular deaths will be further classified into the following categories:
A. Infection
B. Malignancy
C. Pulmonary Failure
D. Gastrointestinal – Death due to GI-related abnormalities, including hepatobiliary disease
E. Renal Failure*
F. Accidental/Trauma
G. Suicide
H. Non Intracranial Hemorrhage (not related to CV surgery/procedure)
I. Other Non-CV (Specify)

* Renal Death is defined as death occurring from complications of renal failure (e.g.hyperkalemia, uremia, acidosis) while a patient receives renal replacement therapy (i.e. chronic dialysis or renal transplantation), or after a patient refuses or a physician withholds such therapy or in cases where dialysis is unavailable.

C. Undetermined Cause of Death
Death in which insufficient data is available to make a reasonable differentiation of cardiovascular or non-cardiovascular cause of death.
