# Supplementary material for: Finerenone in heart failure and chronic kidney disease with type 2 diabetes: FINE-HEART pooled analysis of cardiovascular, kidney and mortality outcomes
Source: Nat Med. 2024 Sep 1;30(12):3758–64. doi: 10.1038/s41591-024-03264-4 (PMC11645272; doi:10.1038/s41591-024-03264-4)
Supplement: Supplementary file 2 — Reporting Summary [file 41591_2024_3264_MOESM2_ESM.pdf]

nature portfolio

Corresponding author(s): Scott D. Solomon, MD

Last updated by author(s): Aug 21, 2024

Reporting Summary

Nature Portfolio wishes to improve the reproducibility of the work that we publish. This form provides structure for consistency and transparency in reporting. For further information on Nature Portfolio policies, see our Editorial Policies and the Editorial Policy Checklist.

Statistics

For all statistical analyses, confirm that the following items are present in the figure legend, table legend, main text, or Methods section.

n/a	Confirmed	
☐	☒	The exact sample size (*n*) for each experimental group/condition, given as a discrete number and unit of measurement
☒	☐	A statement on whether measurements were taken from distinct samples or whether the same sample was measured repeatedly
☐	☒	The statistical test(s) used AND whether they are one- or two-sided<br>*Only common tests should be described solely by name; describe more complex techniques in the Methods section.*
☐	☒	A description of all covariates tested
☐	☒	A description of any assumptions or corrections, such as tests of normality and adjustment for multiple comparisons
☐	☒	A full description of the statistical parameters including central tendency (e.g. means) or other basic estimates (e.g. regression coefficient) AND variation (e.g. standard deviation) or associated estimates of uncertainty (e.g. confidence intervals)
☐	☒	For null hypothesis testing, the test statistic (e.g. *F*, *t*, *r*) with confidence intervals, effect sizes, degrees of freedom and *P* value noted<br>*Give P values as exact values whenever suitable.*
☒	☐	For Bayesian analysis, information on the choice of priors and Markov chain Monte Carlo settings
☒	☐	For hierarchical and complex designs, identification of the appropriate level for tests and full reporting of outcomes
☒	☐	Estimates of effect sizes (e.g. Cohen's *d*, Pearson's *r*), indicating how they were calculated

Our web collection on statistics for biologists contains articles on many of the points above.

Software and code

Policy information about availability of computer code

Data collection	Individual participant data from the 3 trials were recoded and harmonized before addition into the study dataset. Categorical variables were harmonised and all variables computed to the same scale or units of measurement. Variable names were standardized across individual trial datasets. The baseline characteristics of trial participants were extracted and relevant subgroup variables extracted. These were then combined into a dataset with an identifier for randomised treatment and the trial the data was extracted from. Time to event (harmonized to days since randomization) and censoring variables for each of the outcomes listed were also extracted. No specific additional software was used for data collection for this pooled analysis.
Data analysis	Statistical analyses were conducted using STATA version 18.

For manuscripts utilizing custom algorithms or software that are central to the research but not yet described in published literature, software must be made available to editors and reviewers. We strongly encourage code deposition in a community repository (e.g. GitHub). See the Nature Portfolio guidelines for submitting code & software for further information.

Data

Policy information about availability of data

All manuscripts must include a data availability statement. This statement should provide the following information, where applicable:

- Accession codes, unique identifiers, or web links for publicly available datasets
- A description of any restrictions on data availability
- For clinical datasets or third party data, please ensure that the statement adheres to our policy

For each of the 3 clinical trials (FIDELIO-DKD, FIGARO-DKD, and FINEARTS-HF), Bayer (the sponsor) commits to sharing upon request from qualified scientific and medical researchers patient-level clinical trial data, study-level clinical trial data, and protocols. Interested researchers can use www.vivli.org to request access to anonymized patient-level data and supporting documents from clinical studies. Data access will be granted to anonymized patient-level data, protocols and clinical study reports after approval by an independent scientific review panel, with scope and conditions laid out as on https://vivli.org/ourmember/bayer/.

Research involving human participants, their data, or biological material

Policy information about studies with human participants or human data. See also policy information about sex, gender (identity/presentation), and sexual orientation and race, ethnicity and racism.

Reporting on sex and gender	The n (%) of males/females was described overall, by randomized therapy, and by trial population (FIDELIO-DKD, FIGARO-DKD, and FINEARTS-HF). Sex was a prespecified subgroup and treatment effects on the primary endpoint were assessed separately in males and females for evidence of heterogeneity in treatment effect.
Reporting on race, ethnicity, or other socially relevant groupings	The n (%) of each racial category (White, Black, Asian, Other) was described by randomized therapy and by trial population (FIDELIO-DKD, FIGARO-DKD, and FINEARTS-HF). Race was a prespecified subgroup and treatment effects on the primary endpoint were assessed separately by racial category for evidence of heterogeneity in treatment effect.
Population characteristics	Patients with type 2 diabetes and chronic kidney disease with albuminuria (FIDELIO-DKD and FIGARO-DKD trials) and patients with heart failure with mildly reduced or preserved ejection fraction (FINEARTS-HF trial). Mean age was 67±10 years and 35.1% were women.
Recruitment	All participants randomized in each of the 3 trials were considered for this pooled analysis with only patients with critical Good Clinical Practice violations excluded.
Ethics oversight	The trial protocols were approved by ethics committees or institutional review boards at all participating sites and all patients provided explicit written informed consent.

Note that full information on the approval of the study protocol must also be provided in the manuscript.

Field-specific reporting

Please select the one below that is the best fit for your research. If you are not sure, read the appropriate sections before making your selection.

☒ Life sciences ☐ Behavioural & social sciences ☐ Ecological, evolutionary & environmental sciences

For a reference copy of the document with all sections, see nature.com/documents/nr-reporting-summary-flat.pdf

Life sciences study design

All studies must disclose on these points even when the disclosure is negative.

Sample size	All participants validly randomized in each of the 3 trials (FIDELIO-DKD, FIGARO-DKD, and FINEARTS-HF) were considered for this pooled analysis. The 3 trials included 18,991 participants. All efficacy outcomes were analyzed in randomized patients under intention-to-treat principles, while all safety outcomes were analyzed in randomized patients who had taken at least one dose of the study drug.
Data exclusions	Only patients with critical Good Clinical Practice violations were excluded.
Replication	Consistency in treatment effects for the primary and most secondary endpoints was confirmed across each of the 3 individual trial populations, except for the kidney composite endpoint which appeared to be driven by effects observed in FIDELIO-DKD and FIGARO-DKD
Randomization	All participants were randomly allocated 1:1 to finerenone or placebo with initial dosing determined based on kidney function. The initial dose was 10mg for patients with an eGFR <60mL/min/1.73m2, titrated up to a target dose of 20mg once daily as tolerated. Participants with an eGFR ≥60mL/min/1.73m2 were started at the target dose of 20mg once daily. In FINEARTS-HF, participants with an eGFR >60mL/min/1.73m2 were started on 20mg and could be titrated up to 40mg once daily as tolerated, while 20 mg was the target dose for patients with eGFR ≤ 60 ml/min/1.73m2.
Blinding	All 3 trials were double-blind, placebo-controlled randomized clinical trials. Specifically, all investigators remained strictly blinded to treatment arm allocation during data collection and analysis.

Reporting for specific materials, systems and methods

We require information from authors about some types of materials, experimental systems and methods used in many studies. Here, indicate whether each material, system or method listed is relevant to your study. If you are not sure if a list item applies to your research, read the appropriate section before selecting a response.

Materials & experimental systems

n/a	Involved in the study
☒	☐ Antibodies
☒	☐ Eukaryotic cell lines
☒	☐ Palaeontology and archaeology
☒	☐ Animals and other organisms
☐	☒ Clinical data
☒	☐ Dual use research of concern
☒	☐ Plants

Methods

n/a	Involved in the study
☒	☐ ChIP-seq
☒	☐ Flow cytometry
☒	☐ MRI-based neuroimaging

Clinical data

Policy information about clinical studies

All manuscripts should comply with the ICMJE guidelines for publication of clinical research and a completed CONSORT checklist must be included with all submissions.

Clinical trial registration: FIDELIO-DKD [Finerenone in Reducing Kidney Failure and Disease Progression in Diabetic Kidney Disease; NCT02540993]; FIGARO-DKD [Finerenone in Reducing Cardiovascular Mortality and Morbidity in Diabetic Kidney Disease; NCT02545049]); FINEARTS-HF [FINerenone trial to investigate Efficacy and sAfety superioR to placebo in paTientS with Heart Failure; NCT04435626]

Study protocol: The statistical analysis plan for this pooled analysis was prespecified and the protocol was prospectively registered in the International Prospective Register of Systematic Reviews (PROSPERO CRD42024570467).

Data collection: Participants in FIDELIO-DKD were enrolled from September 2015 through June 2018 across 48 countries. Participants in FIGARO-DKD were enrolled from September 2015 through October 2018 across 48 countries. Participants in FINEARTS-HF were enrolled from September 2020 through January 2023 across 37 countries. All 3 trials were global clinical trials with enrollment from academic/ hospital-based or community health care facilities.

Outcomes: The prespecified primary endpoint for FINE-HEART was time to cardiovascular death. All deaths were adjudicated by independent clinical endpoint committees in each of the respective trials included in this pooled analysis. Other prespecified endpoints included a kidney composite endpoint (defined as a sustained decline in eGFR to ≥50% from baseline, sustained decrease in eGFR to <15 mL/ min/1.73m2, end-stage kidney disease, and death due to kidney causes), HF hospitalization, composite of cardiovascular death or HF hospitalization, new-onset atrial fibrillation, major adverse cardiovascular events (a composite of non-fatal myocardial infarction, non-fatal stroke, HF hospitalization, or cardiovascular death), all-cause death, and all-cause hospitalization. The composite of all-cause death or all-cause hospitalization was defined post hoc.

Plants

Seed stocks: *Report on the source of all seed stocks or other plant material used. If applicable, state the seed stock centre and catalogue number. If plant specimens were collected from the field, describe the collection location, date and sampling procedures.*

Novel plant genotypes: *Describe the methods by which all novel plant genotypes were produced. This includes those generated by transgenic approaches, gene editing, chemical/radiation-based mutagenesis and hybridization. For transgenic lines, describe the transformation method, the number of independent lines analyzed and the generation upon which experiments were performed. For gene-edited lines, describe the editor used, the endogenous sequence targeted for editing, the targeting guide RNA sequence (if applicable) and how the editor was applied.*

Authentication: *Describe any authentication procedures for each seed stock used or novel genotype generated. Describe any experiments used to assess the effect of a mutation and, where applicable, how potential secondary effects (e.g. second site T-DNA insertions, mosiacism, off-target gene editing) were examined.*
